# Improved Methods for Acetocarmine and Haematoxylin Staining to Visualize Chromosomes in the Filamentous Green Alga *Zygnema* (Charophyta)

**DOI:** 10.21769/BioProtoc.4768

**Published:** 2023-08-20

**Authors:** Nina Rittmeier, Andreas Holzinger

**Affiliations:** Functional Plant Biology, Department of Botany, University of Innsbruck, Innsbruck, Austria

**Keywords:** Acetocarmine, Haematoxylin, Hydroxyquinoline, Chromosomes, *Zygnema*, Zygnematophyceae, Charophyta, Light microscopy

## Abstract

Genome sizes of *Zygnema* spp. vary greatly, being unknown whether polyploidization occurred. The exact number of chromosomes in this genus is unknown since counting methods established for higher plants cannot be applied to green algae. The massive presence of pectins and arabinogalactan proteins in the cell wall interferes with the uptake of staining solutions; moreover, cell divisions in green algae are not restricted to meristems as in higher plants, which is another limiting factor. Cell divisions occur randomly in the thallus, due to the intercalary growth of algal filaments. Therefore, we increased the number of cell divisions via synchronization by changing the light cycle (10:14 h light/dark). The number of observed mitotic stages peaked at the beginning of the dark cycle. This protocol describes two methods for the visualization of chromosomes in the filamentous green alga *Zygnema*. Existing protocols were modified, leading to improved acetocarmine and haematoxylin staining methods as investigated by light microscopy. A freeze-shattering approach with liquid nitrogen was applied to increase the accessibility of the haematoxylin dye. These modified protocols allowed reliable chromosome counting in the genus *Zygnema*.

Key features

Improved method for chromosome staining in filamentous green algae.

Optimized for the *Zygnema* strains SAG 698-1a (*Z. cylindricum*), SAG 698-1b (*Z. circumcarinatum*), and SAG 2419 (*Zygnema* ‘Saalach’).

This protocol builds upon the methods of chromosomal staining in green algae developed by Wittmann (1965), Staker (1971), and Fujii and Guerra (1998).

Cultivation and synchronization: 14 days; fixation and permeabilization: 24 h; staining: 1 h; image analysis and chromosome number quantification: up to 20 h.

## Background

**Background on chromosome counting in *Zygnema***

Zygnematophyceae are the immediate sister group to land plants, thought to have colonized land 550 million years ago (Wodniok et al., 2011; Leebens-Mack et al., 2019). Data on nuclear DNA content are available for *Zygnema* (Čertnerová, 2021; Feng et al., 2021 and 2023), and genome sizes for different strains of *Zygnema* spp. have been established, but these show great variation (Feng et al., 2023). Chromosomes of *Zygnema* have previously been illustrated by transmission electron microscopy (Bakker and Lokhorst, 1987), although without giving actual numbers. The actual chromosome numbers of *Zygnema* vary drastically, ranging from 14 to 82 (Kurssanow, 1911; Miller 1973; Guiry et al., 2022). Thus, it is unclear if polyploidization has happened. Traditional counting methods established for higher plants are hampered in green algae by pectins [massive homogalacturonan accumulations have been reported by Herburger et al. (2019)] and arabinogalactan proteins in the cell wall. Even though numerous protocols have been established for chromosomal staining in green algae (Godward, 1948; Wittmann, 1965; Prasad and Godward, 1966; Staker, 1971; Gerlach, 1977; Fujii and Guerra, 1998), they still face difficulties handling *Zygnema* spp., as their chromosomes are small, sticky, and mostly only able to be counted in their mid- to late-prophase. Furthermore, conventional staining methods lead to over-staining of the cell wall and DNA-dense areas in the cytoplasm known as *karyoide* in *Zygnema*, Charophyta (Kopetzky-Rechtperg, 1934).

**Modification of the methods from Wittmann (1965), Staker (1971), and Fujii and Guerra (1998)**

**Figure 1.**
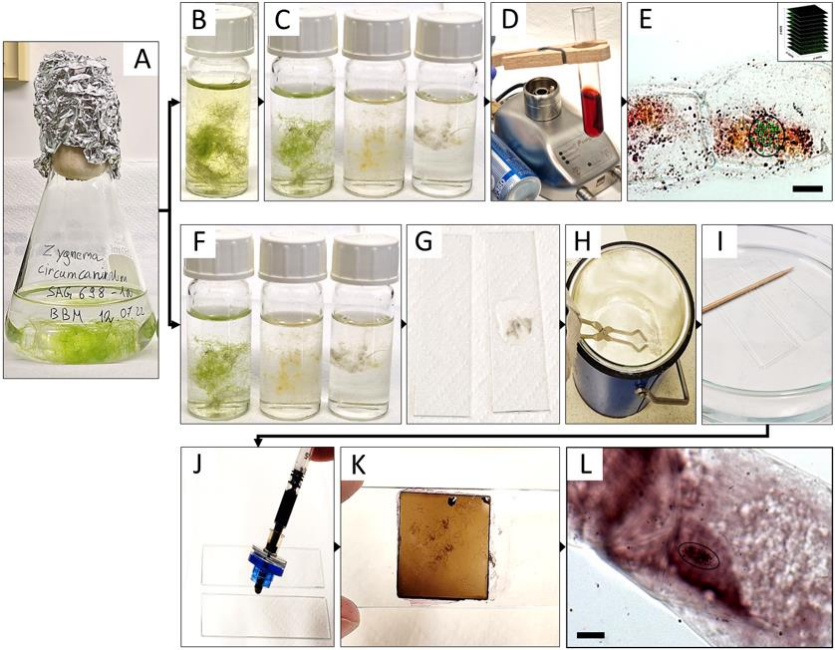
Summary of the experimental procedure for acetocarmine (B–E) and haematoxylin (F–L) staining in *Zygnema circumcarinatum*. (A) Erlenmeyer flask with young culture; (B) 8-hydroxyquinoline prefixation; (C, F) fixation in Carnoy’s fluid, illustrating the gradual bleaching from time 0, 1, and 12 h (from left to right); (D) staining with acetocarmine; (E) projected Z-stack rendered by Helicon Focus software used for counting the chromosomes (circle: chromosomes marked with green crosses in ImageJ; inset: symbolic representation of individual images for Z-stacks); (G) algal biomass mounted in acetic acid and squished between two slides; (H) slides dipped in liquid nitrogen; (I) HCl treatment; (J, K) aceto-haematoxylin-iron alum staining; (L) light microscopic image used for quantifying the number of chromosomes (circle). Scale bars = 10 µm.

For all three *Zygnema* ssp., two different modified staining procedures were used: (1) acetocarmine (Figure 1A–1E) and (2) haematoxylin (Figure 1A, 1F–1L). To achieve full mitotic synchronization in *Zygnema*, a light/dark cycle (10:14 h) was applied following Staker (1971), i.e., with a longer dark period than our standard cultivation cycle (light/dark, 16:8 h).

For the staining of the chromosomes, Staker (1971) used propriocarmine, which was replaced with 1% acetocarmine in the current protocol; also, the *Zygnema* filaments were not chopped or randomized, which would lead to complete destruction. Moreover, prior to the fixation in Carnoy’s fluid, cells were treated with 8-hydroxyquinoline to depolymerize microtubules, resulting in sticky and condensed metaphase chromosomes (Bukhari, 2004). Haematoxylin staining by Wittmann (1965) was used, but the treatment with chloralhydrate and slide heating steps was omitted and replaced with hydrolysis by 5 N HCl (Fujii and Guerra, 1998), resulting in contrast improvement between chromosomes and cytoplasm. Changes only occurred with the algal filaments being placed between two microscopic slides before liquid nitrogen treatment with a freeze-shattering method (Wasteneys et al., 1997); the HCl treatment time was reduced to 10 min to assure the integrity of the Z*ygnema* filaments, which disintegrate after prolonged treatment.

## Materials and reagents

**Biological material**

1. *Zygnema cylindricum* strain SAG 698-1a (Figure 2A; Feng et al., 2021; isolated 1929 by Czurda V.; deposited 1954 by Pringshein E.G.), collected from a ditch at meadow Poselteich (Polenský Rybnik; 50°33′09.7″N 14°40′09.7″E) near Hirschberg (Dosky) in Czech Republic, Europe.
  **Figure 2.**
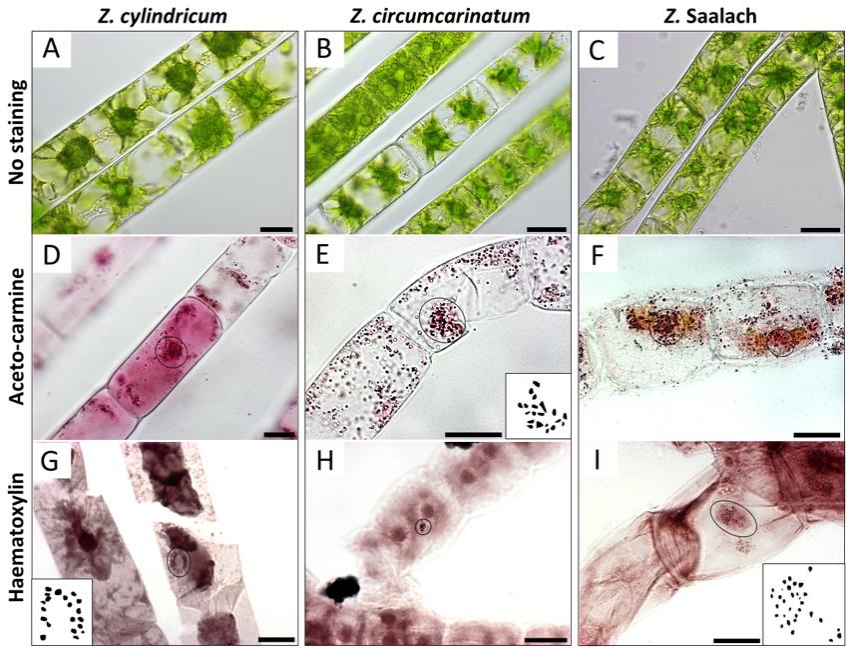
Chromosomes (circles) visualized by light microscopy in different *Zygnema* strains. (A, D, G) *Z. cylindricum*; (B, E, H) *Z. circumcarinatum;* and (C, F, I) *Z.* Saalach. (A, B, C) Living cells without staining; (D, E, F) acetocarmine staining; (G, H, I) haematoxylin staining. Insets in E, G, and I show manual drawings of chromosomes counted with ImageJ; counted chromosome numbers: n = 20 for *Z. cylindricum/Z. circumcarinatum* and n = 30 for *Z.* Saalach. Scale bars = 20 µm.
2. *Zygnema circumcarinatum* strain SAG 698-1b (Figure 2B; isolated 1929 by Czurda V.; deposited 1954 by Pringshein E.G.), collected from a ditch at meadow Poselteich (Polenský Rybnik; 50°33′09.7"N 14°40′09.7″E) near Hirschberg (Dosky) in Czech Republic, Europe. The results of the chromosome counting for this strain have been recently published (Feng et al., 2023)
3. *Zygnema* ‘Saalach’ (SAG 2419; 47°47′8.70″N, 12°56′42.66″E; 440 m above sea level; Figure 2C), collected near Salzburg, Austria (Herburger et al., 2015)

**Reagents**

1. 8-Hydroxyquinoline (Merck, catalog number: 148-24-3)
2. Acetocarmine (Morphisto, catalog number: 10411)
3. Liquid nitrogen
4. 5 N HCl (Merck, catalog number: 258148)
5. Bi-distilled water (A. bidest)
6. Bold’s basal culture medium (BBM), pH 5.5 (Bischoff and Bold, 1963)
7. Glacial acetic acid (Merck, catalog number: A6283)
8. 100% ethanol (Sigma, catalog number: 493546)
9. 45% acetic acid (Merck, catalog number: A6283)
10. Haematoxylin (Merck, catalog number: H9627)
11. Ammonium iron (III) sulfate (Sigma, catalog number: 221260)

**Solutions**

1. 2 mM 8-Hydroxyquinoline (see Recipes)
2. Carnoy’s fluid (see Recipes)
3. Aceto-haematoxylin-iron alum (see Recipes)

## Recipes

1. **2 mM 8-Hydroxyquinoline**
  **Table:** 

Reagent	Final concentration	Quantity
8-Hydroxyquinoline	2 mM	29 mg
A. bidest	100%	1,000 mL
Total		1,000 mL
  This solution can be kept in the dark at room temperature (RT) for up to one year and can last for up to 100 preparations.
2. **Carnoy’s fluid**
  **Table:** 

Reagent	Final concentration	Quantity
Glacial acetic acid	100%	125 mL
Ethanol (absolute)	100%	375 mL
Total		500 mL
  This solution should be prepared immediately before use and can last for up to 50 preparations.
3. **Aceto-haematoxylin-iron alum**
  **Table:** 

Reagent	Final concentration	Quantity
Acetic acid	45%	100 mL
Haematoxylin	0.4%	400 mg
Ammonium iron (III) sulfate	0.1%	100 mg
Total		100 mL
  This solution can be kept at 4 °C for up to half a year and can last for up to 300 preparations.

## Equipment

1. Gas burner
2. Fridge (4 °C)
3. Growth chamber (Panasonic, MLR-352-PE equipped with 2 Panasonic FL40SS·ENW/37 fluorescent tubes)
4. Light microscope [Zeiss Axiovert 200M microscope equipped with a 100×, 1.3 NA objective lens (Carl Zeiss AG)] with a Zeiss high-resolution AxioCam HRm Rev.3 camera
5. Glassware: 250–500 mL Erlenmeyer flask (Analyticsshop.com, ID1121226361), 10 mL glass vials (Merck, 27151), 10 mL test tubes (Merck, Z741001), culture dish (Analyticsshop.com, ø 100 mm), glass jar (for storage)
6. Glass Pasteur pipettes (Analytics Shop.com, BR747715)
7. LLG-Syringe filters, CA, 0.20 m, ø 13 mm (Lab logistics Group, 14140027207)
8. 1 mL Syringe, Omnifix^®^-F (Bio-apo.at, 00569881)
9. 2 mL tubes
10. Lint-free paper
11. Metal rack
12. Microscopic slides and coverslips
13. Wooden clip
14. Pair of fine-pointed tweezers
15. Liquid nitrogen container with lid
16. Leather gloves
17. Long tongs
18. Spray skirt
19. Safety goggles
20. Stopwatch or timer

## Software

1. Helicon Focus (HeliconSoft Ltd.)
2. ImageJ (1.53v)

## Procedure

- **Acetocarmine staining**
  1. Synchronization of mitotic activity
    - Place pure algal biomass into a 250–500 mL Erlenmeyer flask containing 150–250 mL of BBM (Figure 1A).
    - Grow cultures in a light/dark regime of 10:14 h at 20 °C and 50 μmol photons m^-2^·s^-1^ in the light period for two to three weeks to obtain log-phase cultures.
  2. Harvesting of algal biomass
    - Collect algal biomass at the beginning of the dark cycle in the laminar flow hood (to maintain sterility of the cultures).
    - Place the sample with the fine-pointed tweezers into 2 mL tubes containing 1 mL of A. bidest at RT.
    - Fix collected samples immediately after collection.
  3. Fixation
    - Transfer the samples to 10 mL glass vials containing 5 mL of 2 mM 8-hydroxyquinoline, leave at RT for 1–2 h under the fume hood, and then transfer to 4 °C for 1–2 h (Figure 1B).
*Note: The necessary time has to be tested out for each species. For SAG 698-1a and SAG 698-1b, 1 h each was enough, while for SAG 2419, 2 h was needed.*
    - Remove 8-hydroxyquinoline completely with a glass Pasteur pipette and wash the sample with 5 mL of A. bidest three times for at least 1 min under the fume hood.
    - Immediately submerge samples in 5 mL of Carnoy’s fluid and leave at RT for 12 h until all chlorophyll is removed and the samples are visibly bleached (Figure 1C).
  4. Staining
    - Collect the current bleached sample with the fine-pointed tweezers and place in the test tube containing 5 mL of 1% acetocarmine.
    - Hold the test tube with the wooden clip over the gas burner at low flame by not constantly keeping it in the flame and boil the algal biomass in acetocarmine for 5 min under the fume hood (Figure 1D).
    - Pour the acetocarmine-boiled algal biomass into the culture dish. Select the stained filaments with fine-pointed tweezers, place them onto a microscopic slide with a small droplet of acetocarmine, and place a coverslip on top.
  5. Microscopical analysis
    - Visualize the stained chromosomes with a light microscope (Figure 1E).
    - Take 50–100 images per area in Z-direction.
*Note: Either use an automated focus or capture images manually with a distance of 0.2–0.4 µm.*
    - Render stacked models with the software Helicon Focus (HeliconSoft Ltd.).
    - Count the chromosomes with ImageJ.
*Notes:*
      *i. The following tools in ImageJ should be used to process the stacked images; for details, see the following YouTube video by Kevin Foley:* *https://m.youtube.com/watch?v=D1qBaFwuF4E*.
*Process - Subtract Background*
*Image - Adjust - Threshold*
*Process - Binary - Fill Holes*
*Process - Binary - Convert to Mask*
*Process – Binary- Watershed*
*Analyze - Analyze Particles*
*ii. The number of chromosomes counted is based on a minimum of three biological replicates. In Figure 2D, 2E, and 2F, representative samples are illustrated. For each biological replicate, the chromosomes of at least 10 samples were counted (technical replicates).*
- **Haematoxylin staining**
  1. Synchronization of mitotic activity
    - Place pure algal biomass into 250–500 mL tubes containing 200 mL of BBM (Figure 1A).
    - Grow cultures in a light/dark regime of 10:14 h at 20 °C and 50 μmol photons m^-2^·s^-1^ in the light period for at least two weeks.
  2. Harvesting of algal biomass
    - Collect algal biomass at the beginning of the dark cycle.
    - Place the sample with fine-pointed tweezers into 2 mL tubes containing 1 mL of A. bidest at RT.
    - Fix collected samples immediately.
  3. Fixation
    - Place harvested material into the 10 mL glass vial containing 5 mL of Carnoy’s fluid and incubate for 2 h or until sample is completely bleached at RT (Figure 1F).
    - Decant the Carnoy’s fluid and replace with 5 mL of 70% ethanol (samples can be stored in this mixture at 4 °C for up to half a year).
    - Wash samples in 5 mL of A. bidest three times for at least 1 min before transferring the bleached material to a microscopic slide (mount in 45% acetic acid) and squashing with another slide (Figure 1G).
  4. Liquid nitrogen treatment
    - Fill the liquid nitrogen container halfway up with liquid nitrogen (wear safety goggles, leather gloves, and a spray skirt for the whole procedure) and place lid on top.
    - Open the container and use the long tongs to dip the two slides for ten seconds into the liquid nitrogen (Figure 1H).
    - Remove the slides from the liquid nitrogen, pull them apart carefully while still frozen, and let them air dry for at least 30 min.
*Note: The slides can be pulled apart by hand or, if proven difficult, a spatula can be used.*
    - Let the leftover liquid nitrogen evaporate under the fume hood.
  5. HCl treatment
    - Plunge the dried slide with the samples attached into 5 N HCl for at least 10 min under the fume hood (Figure 1I).
    - Remove the slide and let it air dry for another 30 min.
    - Stain the dried slides immediately or keep them in a glass jar at -20 °C for up to half a year.
  6. Staining
    - To stain the material on the microscopic slides, mount it with one droplet of the aceto-haematoxylin-iron alum (Figure 1J) and place a coverslip on top.
*Note: A syringe equipped with a syringe filter is used to minimize the fallout particles of the solution, which could lead to contamination of the sample.*
    - Full saturation is reached after an incubation time of 5 min (Figure 1K); the excessive dye can be removed with a lint-free paper.
  7. Microscopical analysis
    - Visualize the stained chromosomes with a light microscope (Figure 1L).
    - Take up to 100 images per area in Z-direction.
    - Render stacked models with the software Helicon Focus (HeliconSoft Ltd.).
    - Count the chromosomes with ImageJ (see Section A, step 5d).
*Note: The numbers of chromosomes counted are based on a minimum of three biological replicates; in Figure 2G, 2H, and 2I, representative samples are illustrated. Per biological replicate, the chromosomes of at least 10 samples were counted (technical replicates).*
